# Hippocampal atrophy in neurofunctional subfields in insomnia individuals

**DOI:** 10.3389/fneur.2022.1014244

**Published:** 2022-10-17

**Authors:** Yang Yang, Wei Liang, Yongjun Wang, Dechang Peng, Liang Gong, Na Wang, Zhongjie Huang, Weikang Yang

**Affiliations:** ^1^Department of Radiology, Suining Central Hospital, Suining, China; ^2^Shenzhen Mental Health Centre, Shenzhen Kangning Hospital, Shenzhen, China; ^3^Department of Radiology, The First Affiliated Hospital of Nanchang University, Nanchang, China; ^4^Department of Neurology, Chengdu Second People's Hospital, Chengdu, China; ^5^Department of Radiology, Shenzhen Longhua Maternity and Child Healthcare Hospital, Shenzhen, China; ^6^Department of Prevention and Healthcare, Shenzhen Longhua Maternity and Child Healthcare Hospital, Shenzhen, China

**Keywords:** insomnia, hippocampus, voxel-based morphometry, nomogram, mediation

## Abstract

**Objective:**

The aim of this study was to investigate the pattern of volume changes in neurofunctional hippocampal subfields in patients with insomnia and their associations with risk of development of insomnia.

**Methods:**

A total of 120 patients with insomnia (78 females, 42 males; mean age ± standard deviation, 43.74 ± 13.02 years) and 120 good sleepers (67 females, 53 males; mean age, 42.69 ± 12.24 years) were recruited. The left hippocampus was segmented into anterior (L1), middle (L2), and posterior (L3) subregions. The right hippocampus was segmented into top anterior (R1), second top anterior (R2), middle (R3), posterior (R4), and last posterior (R5) subregions. Multivariate logistic regression was used to evaluate the associations of hippocampal volume (HV) of each subfield with the risk of the development of insomnia. Mediation analyses were performed to evaluate mediated associations among post-insomnia negative emotion, insomnia severity, and HV atrophy. A visual easy-to-deploy risk nomogram was used for individual prediction of risk of development of insomnia.

**Results:**

Hippocampal volume atrophy was identified in the L1, R1, and R2 subregions. L1 and R2 volume atrophy each predisposed to an ~3-fold higher risk of insomnia (L1, odds ratio: 2.90, 95% confidence intervals: [1.24, 6.76], *p* = 0.014; R2, 2.72 [1.19, 6.20], *p* = 0.018). Anxiety fully mediates the causal path of insomnia severity leading to R1 volume atrophy with a positive effect. We developed a practical and visual competing risk-nomogram tool for individual prediction of insomnia risk, which stratifies individuals into different levels of insomnia risk with the highest prediction accuracy of 97.4% and an average C-statistic of 0.83.

**Conclusion:**

Hippocampal atrophy in specific neurofunctional subfields was not only found to be associated with insomnia but also a significant risk factor predicting development of insomnia.

## Introduction

Insomnia is one of the most prevalent health complaints worldwide (1) and is characterized by nonrestorative sleep and difficulties in sleep initiation/maintenance and falling asleep (2). Insomnia increases the risk of dementia and is associated with mood disturbances and cognitive function deficits, including attention, memory, decision-making, and executive function (3–8). The hippocampus plays a crucial role in the acquisition, consolidation, and recovery of memory (9, 10). Different hippocampal subfields are responsible for different cognitive functions and are affected by neuropsychiatric conditions to varying degrees (11–13).

Despite a recent increase in studies concerning the association of hippocampal volume (HV) with insomnia, it has not gleaned a consistent finding, and further replication is needed. For example, Riemann et al. found significantly reduced bilateral HV in patients with insomnia compared with good sleepers (14). Joo et al. found that patients with insomnia exhibited bilateral atrophy across all hippocampal subfields (15). Conversely, Winkelman et al. and Spiegelhalder et al. found a lack of HV differences in patients with primary insomnia compared with good sleepers (16, 17). These studies evaluated structural hippocampal atrophy; however, to the best of our knowledge, no studies have performed a comprehensive examination to identify and characterize neurofunctional subfields of HV in patients with insomnia.

Understanding the functional topography of the hippocampus could lead to advances in our understanding of healthy cognitive processing and have transformative implications on understanding how hippocampal-dependent cognitive and emotive processing occurs in disease status in which the hippocampus has been implicated.

The aim of this study was to investigate the pattern of volume changes in neurofunctional hippocampal subfields in patients with insomnia compared with good sleepers. First, we evaluated which hippocampal subfield had volumetric atrophy. Second, multivariable logistic regression analysis was used to evaluate the associations of volume atrophy of neurofunctional hippocampal subfields with the risk of the development of insomnia. Third, we developed a visual competing risk nomogram to evaluate the potential of the classifier to identify high-risk patients with insomnia based on the volume atrophy of neurofunctional hippocampal subfields. Fourth, mediation analyses were performed to evaluate the mediated associations among post-insomnia negative emotions (referring to the consequences of insomnia), insomnia severity, and volume atrophy of neurofunctional hippocampal subfields.

## Materials and methods

### Subjects

A total of 120 patients with insomnia (78 females, 42 males; mean age ± std, 43.74 ± 13.02 years) and 120 age-, sex-, and education-matched good sleepers (good sleepers; 60 females, 60 males; mean age, 42.69 ± 12.24 years) were recruited from the Department of Psychiatry in the hospital and community advertising (Table 1). This study was approved by the Medical Research Ethical Committee of Chengdu Second People's Hospital, Suining Central Hospital, and the First Affiliated Hospital of Nanchang University in accordance with the Declaration of Helsinki.

**Table 1:** Demographic characteristics of insomnia group and good sleepers.

	**Insomnia group**	**Good sleepers**	**t/χ2 value**	***p*** **value**
Mean age, year	43.74 ± 13.02	42.69 ± 12.24	0.64	0.5
Sex (Male, Female)	120 (42, 78)	120 (53, 67)	2.11^#^	0.2
Duration of insomnia, year	5.62 ± 6.16	N/A	N/A	N/A
Pittsburgh Sleep Quality Index (PSQI)	14.37 ± 2.21	2.78 ± 0.86	51.00	<0.001
Self-Rating Anxiety Scale (SAS)	47.76 ± 9.41	30.44 ± 8.70	13.66	<0.001
Self-Rating Depression Scale (SDS)	50.99 ± 9.06	32.22 ± 5.79	17.63	<0.001

Data are mean ± standard deviation values;

# chi-square value.

N/A, not applicable.

Patients with insomnia met the relevant diagnostic criteria of the International Classification of Sleep Disorders, Third Edition, had a Pittsburgh Sleep Quality Index (PSQI) score > 5, aged between 18 and 65 years, age of insomnia onset under 50 years, and completed a sleep diary for more than 1-week duration. Furthermore, patients with insomnia had to report a total sleep time ≤7 h and one of the following: (a) sleep onset latency >45 min, (b) wake after sleep onset >45 min, and (c) total wake time during the sleep period (sleep latency + wake after sleep onset) >60 min. All patients with insomnia should have at least 1 month of difficulty falling asleep, maintaining sleep, or early wakening; 23 patients with insomnia (7 males and 16 females) were not first-time visitors and had taken hypnotic medications or psychoactive medications for more than 2 weeks prior to this study.

All good sleepers met the following criteria: had good sleeping habits, reported a sleep onset of <30 min, and were not easily wakened or experience morning awakening symptoms, did not consume any stimulant, hypnotic, or psychoactive medications during or for ≥3 months prior to this study, and had a PSQI score <5, a Hamilton Depression Rating Scale (HAMD) score <7, and a Hamilton Anxiety Rating Scale (HAMA) score <7.

The exclusion criteria for all subjects comprised pathological brain magnetic resonance imaging (MRI) findings; inborn or other acquired diseases; foreign implants in the body; body mass index (BMI) >32 or <19.8; present or past psychiatric or neurological disorders, substance dependency, or substance abuse (including heroin, nicotine, or alcohol addiction; hypnotic medication was not included); recent experience of swing shift, night shift, or other shift work; any history of sleep disorders, such as hypersomnia, parasomnia, sleep-related breathing disorder, sleep-related movement disorder, or circadian rhythm sleep disorder; any history of significant head trauma or loss of consciousness >30 min; current smoking of more than 10 cigarettes per day; and consumption of >2 caffeinated beverages or potent tea per day.

To evaluate their sleep status, all subjects were asked to wear a Fitbit Flex tracker (http://help.fitbit.com) (18). These data were primarily used to verify sleep-wake diary information and were not used for an independent assessment of inclusion and exclusion criteria. Volunteers were asked to complete a number of questionnaires, including the PSQI, Self-Rating Anxiety Scale (SAS), and Self-Rating Depression Scale (SDS). An experienced psychiatrist evaluated the life histories of patients with insomnia with the Diagnostic and Statistical Manual of Mental Disorders, version 5 (DSM-V) for the presence of psychiatric disorders, as well as an unstructured clinical interview for medical and sleep disorder history. All volunteers participated voluntarily and signed an informed consent form.

### MRI

MRI scans were performed on 3-Tesla MR scanners (Trio, Siemens, Erlangen, Germany). High-resolution T1-weighted anatomical images were acquired with a three-dimensional spoiled gradient-recalled sequence in a sagittal orientation: 176 images (repetition time = 1,900 ms, echo time = 2.26 ms, thickness = 1.0 mm, gap = 0.5 mm, acquisition matrix = 256 × 256, field of view = 250 mm × 250 mm, flip angle = 9^0^) were obtained. Then, a total of 240 functional volumes (repetition time = 2,000 ms, echo time = 30 ms, thickness = 4.0 mm, gap = 1.2 mm, acquisition matrix = 64 × 64, flip angle = 90°, and field of view = 220 mm × 220 mm) covering the whole brain were obtained. A simple questionnaire was administered immediately after the MRI scan to determine whether subjects were awake during the MRI scan. Data from the subjects who were asleep during the MRI scan were excluded.

### Data preprocessing

The CAT12 toolbox (http://dbm.neuro.uni-jena.de/cat12/) was used to perform the data preprocessing as in our previous study (19). Structural images were segmented into gray matter (GM), white matter (WM), and cerebrospinal fluid (CSF) using the Diffeomorphic Anatomical Registration Through Exponentiated Lie algebra (DARTEL) segmentation procedure. The data were spatially normalized using the East Asian brain template in the Montreal Neurological Institute (MNI; http://www.mni.mcgill.ca/) space. The segmented data were modulated and smoothed using a Gaussian kernel of 6 × 6 × 6 mm^3^ full-width at half-maximum.

### Definition of left hippocampal subregions

The hippocampal subregions were based on a recent data-driven characterization that revealed a subspecialization in the hippocampus using coactivation-based parcellation (20). The left hippocampus was segmented into three subregions, namely, the anterior subregion (L1), involved in cognitive and emotional processes; the middle subregion (L2), involved in cognitive processes; and the posterior subregion (L3), involved in the perceptual function. The right hippocampus was segmented into five subregions, namely, the top anterior subregion (R1), mostly involved in emotional processes; the second top anterior subregion (R2), mostly involved in cognitive processes; the middle subregion (R3), mostly involved in emotional processes; the posterior subregion (R4), involved in emotional and cognitive processes; and the last posterior subregion (R5), involved in emotional processes.

### Statistical analyses

Comparisons of demographic factors and questionnaires between the insomnia group and good sleepers were performed using two-sample *t*-tests. Chi-square (χ^2^) tests were used for categorical data. Statistical analysis was performed using IBM SPSS 21.0. Data are presented as mean ± standard deviation. *p* < 0.05 was considered statistically significant.

Logistic regression was used to evaluate the associations of hippocampal subfields with the risk of insomnia. Univariate and multivariate models were applied to evaluate the odds ratio (OR) and 95% confidence intervals (95% CIs). Three schemes were performed in the logistic regression analysis: (1) model 1–unadjusted; (2) model 2–adjusted for age, sex, and total intracranial volume (TIV); (3) model 3–further adjusted for L1, R1, R2, and R3 volumes. We developed a visual easy-to-deploy risk-nomogram tool for identifying high-risk patients with insomnia based on the atrophy of the hippocampal subfields. Model performance was described using bootstrap bias-corrected concordance probabilities (C-statistics). Mediation analyses were performed to evaluate the mediated associations of post-insomnia negative emotion, insomnia severity, and hippocampal atrophy.

## Results

### Sample characteristics

The demographic characteristics of the study sample are presented in Table 1. The groups did not significantly differ in sex distribution (*p* = 0.2) and age (*p* = 0.5). The patients with insomnia had significantly higher PSQI, SAS, and SDS scores than the good sleepers. The average duration of insomnia was 5.62 ± 6.16 years.

### Hippocampal volumes

The details of the volumes of the bilateral hippocampal subfields are presented in Table 2. There were no significant differences in the GM volume (GMV)/TIV, WM volume (WMV)/TIV, and CSF/TIV between the insomnia group and good sleepers. Two-tailed *t*-tests uncovered a significant 2.44% decrease in the left HV (L_HV) and a 3.4% decrease in the right HV (R_HV) of insomnia group vs. good sleepers (left: 1.60 ± 0.16 vs. 1.64 ± 0.15 mm^3^; right: 2.84 ± 0.26 vs. 2.94 ± 0.26 mm^3^). However, there were no significant differences in the L_HV/TIV (*p* = 0.2) and R_HV/TIV (*p* = 0.7) between the insomnia group and good sleepers.

**Table 2:** Altered hippocampal subfield volumes in patients with insomnia and good sleepers.

**Region of interest**	**Patients with insomnia**	**Good sleepers**	***P*** **value**
L1	0.61 ± 0.07	0.65 ± 0.06	<0.001
L2	0.51 ± 0.05	0.51 ± 0.05	0.3
L3	0.48 ± 0.06	0.47 ± 0.05	0.4
R1	0.66 ± 0.07	0.71 ± 0.07	<0.001
R2	0.55 ± 0.06	0.58 ± 0.06	<0.001
R3	0.61 ± 0.06	0.63 ± 0.06	0.01
R4	0.51 ± 0.05	0.51 ± 0.05	0.5
R5	0.51 ± 0.06	0.50 ± 0.05	0.4
L1/TIV, ‰	0.42 ± 0.04	0.43 ± 0.04	0.037
L2/TIV, ‰	0.35 ± 0.03	0.34 ± 0.03	0.041
L3/TIV, ‰	0.33 ± 0.04	0.31 ± 0.04	<0.001
R1/TIV, ‰	0.45 ± 0.04	0.47 ± 0.04	0.01
R2/TIV, ‰	0.37 ± 0.03	0.39 ± 0.03	0.007
R3/TIV, ‰	0.42 ± 0.03	0.41 ± 0.03	0.4
R4/TIV, ‰	0.35 ± 0.03	0.34 ± 0.03	0.006
R5/TIV, ‰	0.35 ± 0.04	0.33 ± 0.03	<0.001
Left hippocampal volume	1.60 ± 0.16	1.64 ± 0.15	0.047
Right hippocampal volume	2.84 ± 0.26	2.94 ± 0.26	0.003
Left hippocampal volume/TIV, ‰	1.10 ± 0.09	1.10 ± 0.09	0.2
Right hippocampal volume/TIV, ‰	1.90 ± 0.15	1.90 ± 0.14	0.7
GMV/TIV, %	44.65 ± 2.21	44.72 ± 2.54	0.8
WMV/TIV, %	34.71 ± 1.50	34.68 ± 1.63	0.9
CSF/TIV, %	20.64 ± 2.61	20.60 ± 2.60	0.9

Data are presented as mean ± SD. L1, L1 volume. L1/TIV,

‰ , TIV volume was divided by L1 volume.

TIV, total intracranial volume.

In the insomnia group, hippocampal atrophy was identified in four subfields. The insomnia group had significantly lower volumes in the L1, R1, R2, and R3 subfields than the good sleepers, and the groups did not significantly differ in the other four hippocampal subfields. The insomnia group had a significantly lower volume proportion of L1/TIV, R1/TIV, and R2/TIV and a higher volume proportion of L2/TIV, L3/TIV, R4/TIV, and R5/TIV, than the good sleepers. However, the groups did not significantly differ in R3/TIV.

### OR risk of hippocampal subfields for insomnia group

The multivariate logistic regression model was used to evaluate whether volume atrophy in particular hippocampal subregions was associated with a higher risk of insomnia. In the final model, we adjusted for age, sex, TIV, L1 volume, R1 volume, R2 volume, and R3 volume (Table 3). The subjects with a L1 (OR [95% CI], 2.90 [1.24, 6.76], *p* = 0.014) and R2 (2.72 [1.19, 6.20], *p* = 0.018) atrophy were predisposed to a higher risk of insomnia compared with those with normal HV.

**Table 3:** Odds ratios and 95% CIs for the association between hippocampal subfields and insomnia using logistic regression analysis.

**Variables**	**Model 1**		**Model 2**		**Model 3**	
	**RR (95% CI)**	***P*** **value**	**RR (95% CI)**	***P*** **value**	**RR (95% CI)**	***P*** **value**
L1	2.97 (1.72–5.11)	<0.001	2.38 (1.24–4.58)	0.009	2.90 (1.24–6.76)	0.014
R1	2.29 (1.33–3.97)	0.003	1.60 (0.83–3.08)	0.2	0.69 (0.27–1.76)	0.4
R2	2.97 (1.71–5.18)	<0.001	2.29 (1.21–4.35)	0.011	2.72 (1.19–6.20)	0.018
R3	1.74 (1.03–2.92)	0.037	1.06 (0.55–2.03)	0.9	0.47 (0.20–1.09)	0.1

Model 1 Unadjusted.

Model 2 Analyses were adjusted for age, sex, and TIV.

Model 3 Analyses were further adjusted for L1, R1, R2, and R3.

TIV, total intracranial volume.

### Development of a competing-risk nomogram

**Figure 1:**
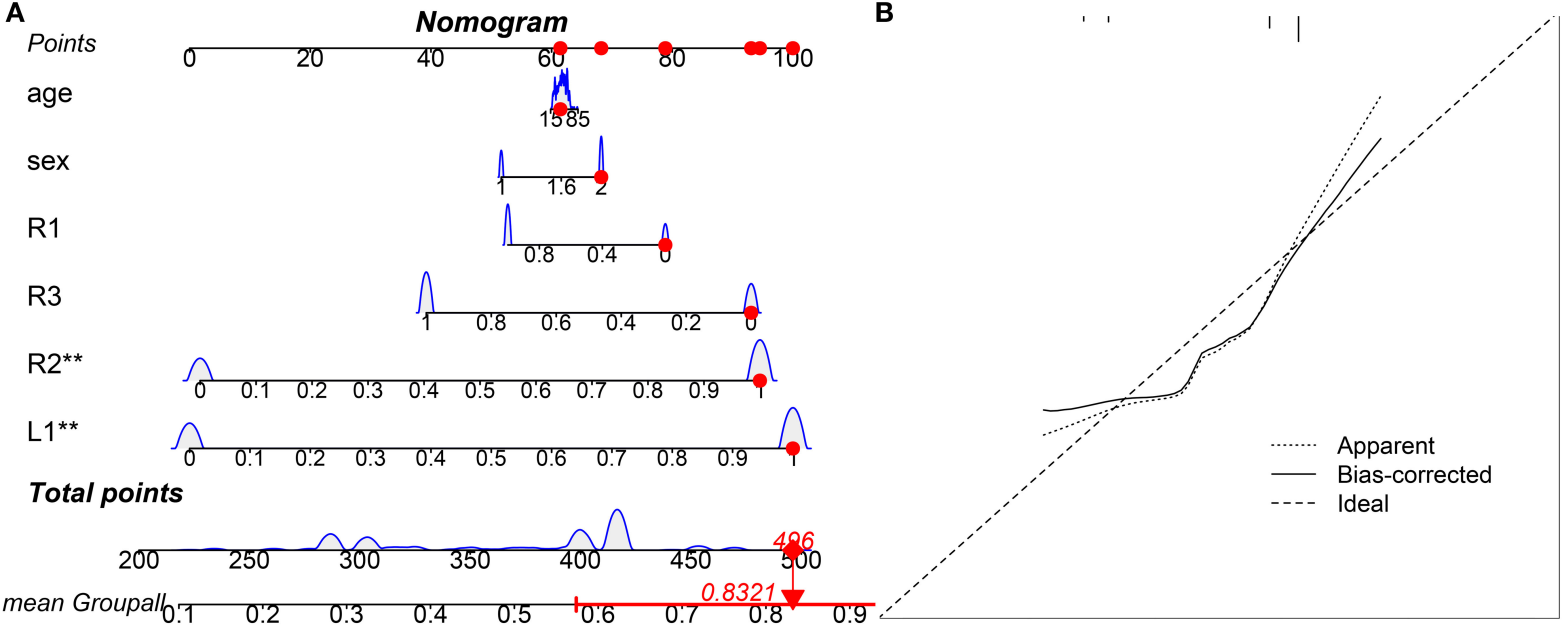
Visual competing risk-nomogram tool for individual prediction of insomnia. The TrackPoint shows an example of one subject for his/her point score and corresponding risk of development of insomnia. For this subject, his/her total point score is 496, and the corresponding risk of development of insomnia is 83.21%. **(A)** Competing-risk nomogram; **(B)** Agreement calibration curve.

Hippocampal atrophy was identified in the L1, R1, R2, and R3 subfields, these factors were incorporated into the multivariate analysis, and a competing-risk nomogram was constructed based on these four factors (Figure 1). The total point score ranged from 200 to 500, which stratified individuals into different levels of insomnia risk (Figure 1A). The prediction accuracy of the competing-risk nomogram reached 97.4%, with an average C-statistic of 0.83. The calibration curve showed good agreement between the nomogram and actual observations (Figure 1B).

### Mediating causality analysis

**Figure 2:**
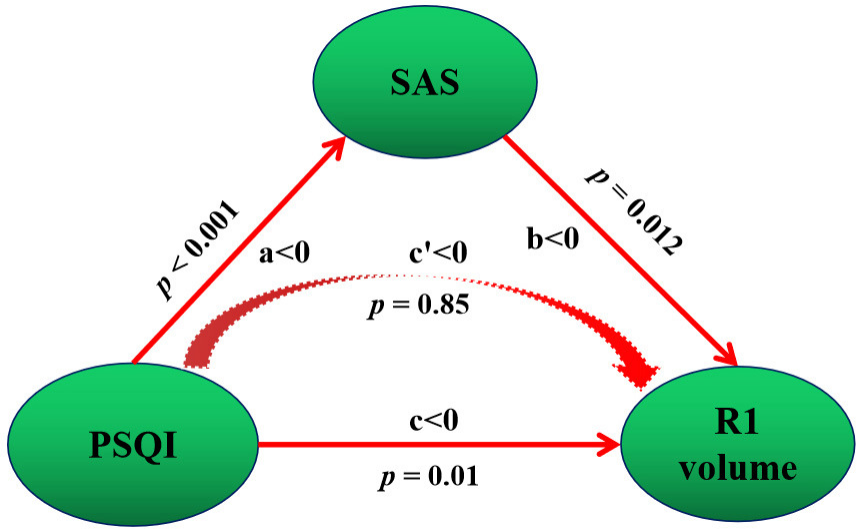
Mediation association of post-insomnia negative emotion, insomnia severity, and hippocampal atrophy. Negative emotion refers to SAS. PSQI refers to insomnia severity. Total PSQI score was negatively correlated with R1 volume, which indicates a positive correlation with R1 volume atrophy. SAS score was negative acting in the causal path of PSQI leading to R1 volume, that is, SAS score was positive acting in the causal path of PSQI leading to R1 volume atrophy. Positive and negative mediation acting was determined by the value of *c* and *c*′. In our study, *c*′ < 0, this means a negative mediation acting in the causal path of PSQI leading to R1 volume increase. This is, a positive acting in the causal path of insomnia severity (PSQI score) leading to R1 atrophy (negative *c*′ < 0 and R1 volume increase = positive acting to R1 atrophy). a, the direct effect from PSQI to SAS; b, the direct effect from SAS to R1 volume; c, the direct effect from PSQI to R1 volume. p, p value for the mediation effect.

We studied whether anxiety and depression were intermediaries in the causal path of insomnia severity and hippocampal atrophy. The mediation model suggested that the SAS score was positive acting in the causal path of the PSQI leading to R1 volume atrophy (Figure 2). In the mediation model, the average direct effect was not significant (*p* = 0.82), while the average causal mediation effect was significant (*p* = 0.012). Together, these results indicate that anxiety fully mediates the causal path of insomnia severity leading to R1 volume atrophy with a positive effect.

## Discussion

In this cross-sectional study with a relatively large sample size, three main novel findings are worth noting. First, hippocampal atrophy was identified in three subfields, including the L1, R1, and R2 subfields, in patients with insomnia after controlling for the TIV. These neurofunctional subfields are mainly associated with negative emotions. Second, L1 and R2 atrophy predisposed individuals to an ~3-fold higher risk of insomnia. Third, PSQI scores were negatively correlated with R1 volume, and the anxiety fully mediates the causal path of insomnia severity leading to R1 volume atrophy on positive effect. Fourth, we developed a practical and visual competing risk-nomogram tool for individual prediction of risk for developing insomnia based on the atrophy of hippocampal subfields, which stratified individuals into different levels of insomnia risk with the highest prediction accuracy of 97.4% and an average C-statistic of 0.83.

Sleep deprivation has been shown to reduce cognitive function, which was associated with ultrastructure damage and pyramidal neuron loss in the hippocampus (21). In addition, increased stress-related cortisol has been found in some studies evaluating patients with insomnia compared with good sleepers (22, 23). The hippocampus is more vulnerable to long-term stress because it contains more glucocorticoid receptors than other brain regions (24). One study demonstrated that increased cortisol levels can cause changes in hippocampal structures in other psychiatric disorders (25). In our study, we found that insomnia did not appear to be associated with hippocampal atrophy after controlling for TIV, but structural atrophy in some specific hippocampal subfields was identified. These findings support the findings of the structural vulnerability of specific neurofunctional hippocampal subfields to insomnia.

Numerous studies have confirmed that sleep loss alters normal affective processing and biased perception, memory, and emotions toward negative aspects of experience. For example, de Almondes et al. reported dysfunction of executive function and facial emotion recognition of negative emotions (fear and sadness) in patients with insomnia (26). Kyle et al. found that patients with insomnia had a significantly lower ability to identify the emotional intensity of sadness and fearful expressions than good sleepers (27). In our study, the bilateral hippocampus was segmented into multiple neurofunctional subfields corresponding to different functions, such as the processes of memory consolidation and emotional regulation. The L1 and R1 have been shown to be mostly involved in emotional processes, and the R2 has been shown to be mostly involved in cognitive processes (20). Our study suggests that the L1 and R2 atrophy increased the risk of developing insomnia ~3-fold, and negative emotion was positively acting in the causal path of insomnia severity leading to R1 atrophy. These findings suggest that insomnia is associated with emotional and cognitive processing disorders.

The major strengths of this study are the relatively large sample size and systematic analysis methods. However, there are several limitations that should be addressed (28). First, there were no measures of cognitive function in this study. It is difficult to draw a defined conclusion about the potential relationships between the HV subfields and cognitive function. Second, the insomnia group had clinically significant scores on the HAMA and HAMD scales, which could have potentially confounded the results. Although anxiety, depression, and insomnia are comorbidities, future studies should recruit a larger sample size to explore the functional connectome differences in patients with insomnia with or without anxiety and depressive symptoms. Third, our findings were limited by the use of the Fitbit Flex tracker to monitor sleep quality (18). Although we cannot provide direct evidence to prove whether the Fitbit tracker provides a valid and reliable measure of objective sleep, we compared some patients' data between the Fitbit and polysomnography (PSG) outcomes and found that the results were similar. In fact, our sample was screened to exclude individuals with medical or psychiatric disorders that may affect sleep, and the diagnosis of insomnia mainly depended on the experience of senior physicians who have been working in the field for more than 20 years. Fourth, the sample bias of women oversampled in the insomnia group may be affected by hormonal background and other potential factors. Future studies should consider gender differences. Fifth, our study is limited to the cross-sectional design and therefore the causal relationship between insomnia and atrophy of hippocampal subfields.

In conclusion, our study identified HV atrophy among hippocampal neurofunctional subfields in the insomnia group. These findings could broaden our understanding of insomnia and help individuals identify their potential risk profile.

## Data availability statement

The original contributions presented in the study are included in the article/supplementary material, further inquiries can be directed to the corresponding author.

## Ethics statement

This study was approved by the Medical Research Ethical Committee of Chengdu Second People's Hospital, Suining Central Hospital and the First Affiliated Hospital of Nanchang University in accordance with the Declaration of Helsinki. The patients/participants provided their written informed consent to participate in this study.

## Author contributions

ZH and WY conceived and designed the whole experiment. YY, WL, YW, and NW took responsibility for the integrity of the data and the accuracy of the data analysis. DP and LG collected the data. YY wrote the main manuscript text. YY, WL, YW, ZH, and WY took responsibility for the statistical data analysis and the critical interpretation of the data. All authors contributed to the final version of the manuscript and have read and approved the final manuscript.

## Conflict of interest

The authors declare that the research was conducted in the absence of any commercial or financial relationships that could be construed as a potential conflict of interest.

## Publisher's note

All claims expressed in this article are solely those of the authors and do not necessarily represent those of their affiliated organizations, or those of the publisher, the editors and the reviewers. Any product that may be evaluated in this article, or claim that may be made by its manufacturer, is not guaranteed or endorsed by the publisher.
